# Living Death at the Intersection of Necropower and Disciplinary Power: A Qualitative Exploration of Racialised and Detained Groups in Australia

**DOI:** 10.1007/s10612-022-09623-2

**Published:** 2022-04-23

**Authors:** Samantha O’Donnell

**Affiliations:** School of Social and Political Sciences, The University of Melbourne, Melbourne, Australia

**Keywords:** Necropower, Disciplinary power, State violence, Living death, Immigration Detention, Prison, Indigenous incarceration, Racisms

## Abstract

This article challenges state-sponsored violence in Australia by exploring the experiences of young Indigenous people in youth detention and refugees in immigration detention in Australia as a form of living death. This article examines how this living death manifests by qualitatively analysing publicly accessible first-hand accounts from Indigenous young people about their experiences of youth imprisonment and from refugees about their experiences of immigration detention onshore and offshore. The findings suggest that when necropower and disciplinary power intersect four overlapping expressions of violence emerge: structural violence, epistemic violence, physical violence and brutality, and disciplinary violence. It is the complex overlapping of these multiple forms of harm that creates an experience of living death. In privileging the voices of young Indigenous people and refugees, this article also recognises their continued refusal of past and present colonial structures and the associated violence of carceral spaces.

## Introduction

Brutal images of hoods, shackles and physical abuse towards Indigenous^1^ young people in detention, broadcast nationally in Australia on 25 July 2016 (Four Corners 2016), fractured perceptions of Australia as an egalitarian nation. However, this brutal violence does not exist in isolation. The harms experienced by Indigenous people in prison are insidiously similar to the harms experienced by refugees^2^ imprisoned in immigration detention. These are forms of harm that also sit firmly within past and continuing colonial structures (Anthony 2018; Boochani 2020; Dehm 2020; Giannacopoulos and Loughnan 2020; Pugliese 2008). In ‘refusal’ of this violence (McGranahan 2016), Indigenous people and refugees have a strong and continuing history of resistance and survival. As I establish in this article, a number of criminologists and other critical scholars have also sought to challenge this violence through critical assessment. By using existing theoretical perspectives to reconceptualise the experiences of these two groups in Australia as a *living death* (Mbembe 2019), I build on this critical scholarship. In so doing, I broaden the theoretical tools available to criminologists to challenge state-sanctioned violence in Australia.

I first examine disciplinary power and explore how carceral spaces *operationalise* multiple and extreme forms of violence (Foucault 1975). I focus on Foucault’s (1975) conception of the ‘carceral archipelago’ and affirm his assertion that the acceptable level of punishment and discipline is lowered within carceral spaces. I then explore necropower as the ‘subjugation of life to the power of death’ (Mbembe 2003: 39). I highlight how the operation of this form of power *justifies* exposing racialised groups to violence (Mbembe 2019: 92). In exploring necropower’s reliance on the racist rhetoric of ‘fictionalized enemies’ and ideas of ‘emergency’ and ‘exception’, I examine how this justification process can occur (Mbembe 2019: 70). Finally, I turn to existing scholarship to highlight how this overlapping process of *justification* and *operationalisation* subjects Indigenous people and refugees to multiple forms of violence. It is this intersecting web of harm that I argue amounts to, what Mbembe (2019) describes as, a *living death.*

To evidence and explore how this living death manifests in Australia, I qualitatively examine the experiences of Indigenous young people in prison and refugees in immigration detention in Australia. I rely on publicly accessible first-hand accounts of immigration detention and prison as told by Indigenous young people and refugees between 2005 and 2018. In relation to Indigenous incarceration, the sample includes: (1) twenty-two statements of evidence (and one verbal transcript) from Indigenous young people about their experiences of youth detention tendered to the *Royal Commission and Board of Inquiry into the Protection and Detention of Children in the Northern Territory* (NT Royal Commission) (2017), and (2) where available, the young peoples’ verbal evidence in corresponding transcripts. In relation to immigration detention, the sample includes: (1) *No Friend but the Mountains* by Behrouz Boochani (2018), an autobiographical account of detention on Manus Island, and (2) eighteen testimonies of offshore and onshore detention from the Behind the Wires collection *Stories from detention: They Cannot Take the Sky*, edited by Green et al. (2017).

There are two sample limitations. First, as the sample relies on young peoples’ experiences of youth detention in the Northern Territory, it is not representative. The sample does not explore the experiences of young Indigenous people incarcerated elsewhere in Australia, nor the experiences of incarcerated Indigenous adults. Second, while the sample includes first-hand accounts of onshore detention, it is weighted towards first-hand accounts of detention on Manus Island, Nauru and Christmas Island. The sample is, therefore, not as representative of refugees’ experiences in onshore immigration detention centres on mainland Australia.

It is also important to acknowledge that I am a white, middle-class, researcher who has been advantaged by the Australian settler-colonial state (Kwaymullina 2016: 442). I have benefitted from colonialism and its Indigenous dispossession (Kwaymullina 2016: 442; Parmar 2018: 194). Although this article cannot be separated from my position of privilege, I recognise Indigenous sovereignty and acknowledge that Australia is a settler-colonial state whose existence is rooted in the dispossession and persecution of Indigenous people (Cunneen 2009: 211). I adopt a decolonising perspective that privileges traditionally marginalised voices through narrative analysis (Lewis and Adeney 2014; Rigney 2006). I thematically analyse the data by coding within different categories but also ensure that the voices of the Indigenous young people and refugees that I rely on are amplified.

Through my analysis and findings, I show that young Indigenous people in prison and refugees in immigration detention in Australia experience four interconnecting forms of violence: structural violence, epistemic violence, physical violence and brutality, and disciplinary violence. I argue that it is the co-operation and overlapping of these forms of harm that creates a ‘death-in-life’ (Mbembe 2019: 75). In exploring first-hand accounts through the lens of living death, I also suggest that this harm is state created. In so doing, I move away from explanations that rely on ‘singular subjects and their attendant pathologies’ (Perera and Pugliese 2021: 74) and towards structural explanations that focus on ‘interlocking’ systems of oppression (Bhatia 2020a; Tofighian 2020). By centralising Indigenous and refugee voices, I acknowledge the continuing resistance and survival of these peoples and encourage critical criminological challenges to the continuing state violence.

### Intersections Between Disciplinary Power and Necropower

In this article, I merge aspects of disciplinary power and necropower to explore how harm is produced at the intersection of racism and carcerality, where incarcerated and racialised groups are exposed to multiple and overlapping forms of violence. In focusing on disciplinary power, I limit my examination to Foucault’s (1975: 297) ‘carceral archipelago’ and the emergence of prisons as institutions that discarded the previous boundaries between ‘confinement, judicial punishment and institutions of discipline’. As penal punishment fused with disciplinary regulation, a network of expanding carceral institutions transported a ‘penitentiary technique’ across society at large (Foucault 1975: 297–298). I rely on what Foucault (1975: 301) terms the most significant consequence of this ‘carceral archipelago’, specifically, that punishment became ‘natural and legitimate’ and as a result society’s ‘tolerance to penality’ increased. Prisons are, thus, seen as an ‘additional degree in the intensity of a mechanism’ that operates across society at large (Foucault 1975: 302).

The position I take is arguably different to the position of Blagg and Anthony (2019), two decolonial criminologists, who de-emphasise Foucault’s focus on the prison in the colonial context of Australia. Although I agree that the prison as the ‘apex’ of control requires tempering in Australia (Blagg and Anthony 2019: 160), the alternative focus on camps depicts these spaces as a ‘zone of indistinction’ where any semblance of law is permanently ‘suspended’ (Agamben 1998: 25). As Agamben (1998: 180–184) establishes, individuals targeted in camps are so stripped of all ‘rights and prerogatives that no act committed against them could appear as a crime’. In prisons, unlike camps, it is the appearance of legality and legitimate punishment that enables extreme forms of violence rather than the suspension of law. Carceral spaces operate under a veneer of legitimate penality and, thus, while state killing is only sometimes justified, punishment that is both *within or outside* the scope of the law is more easily depicted ‘to be free of all excess and all violence’ (Foucault 1975: 302). This distinction leads me to retain a focus on prisons in this article, where violence is more easily shrouded in the guise of penitentiary punishment.

This aspect of disciplinary power is usefully framed by Achille Mbembe’s work on *Necropolitics* (2019). Mbembe (2019) critiques what Foucault (1978: 138) labels biopower, the societal shift away from the traditional sovereign power ‘to take life or let live’ and towards the contemporary sovereign ability to ‘foster life or disallow it to the point of death’ (Foucault 1978: 138–138). Mbembe (2019: 92) argues that biopower does not sufficiently explore death and labels the operation of biopower which enables death as necropower: necessarily ‘the subjugation of life to the power of death’. Mbembe (2019: 66, 71) suggests that the right to ‘expose to death’, which Foucault argues emerged in its ‘most complete’ form during the Holocaust, cannot be divorced from plantation slavery, colonial occupation and the apartheid system. Racism is, therefore, central to necropower (Bhatia 2020b). In contexts of racialised exposure to death, Mbembe (2019: 75, 91) explains that necropower ‘refers and appeals [to]…[and] also labours to produce… exceptions, emergencies, and fictionalized enemy’.

Mbembe (2019: 91) highlights how this racist logic can expose groups to actual death as well as a *living death* marked by ‘unfreedom’ and ‘pain’. Mbembe (2019: 21) explores the ‘death-in-life’ experienced by slaves kept in a permanent ‘*state of injury,* in a phantom-like world of horrors and intense cruelty and profanity’, alongside individuals in ‘late-modern occupation’ and their experiences of domination, ‘humiliation’, physical violence and ‘shame’. Mbembe (2019: 91–92) suggests that when such groups are ascribed with ‘the status of the *living dead’,* the ‘discipline of life and the necessities of hardship…are marked by excess’. In other words, such groups are exposed to ‘terror’, ‘pain’ and ‘unfreedom’ (Mbembe 2019: 91). Although necropolitics encompasses much more than this concept of living death, this state of permanent suffering is of particular significance in the carceral context of Australia.

In this article, I show how necropower’s racist logics expose Indigenous people and refugees to violence in Australia. Violence that is justified by the ‘other’ status imparted on these groups and the rhetoric of ‘emergency’ and ‘exception’ (Mbembe 2019: 70). Carceral spaces then operationalise multiple and intersecting forms of violence that are more easily disguised as legitimate punishment (Foucault 1975). I, therefore, argue that when racism and penitentiary punishment intersect racialised groups are *justifiably* exposed to multiple and coalescing forms of violence that are *operationalised* by the heightened penitentiary techniques that are ‘legitimately’ operating within carceral spaces. In making this argument, I explore the Australian propensity to incarcerate refugees and Indigenous people, and its violent consequences.

## The Australian Context

### Incarceration

Indigenous people are disproportionately detained in carceral spaces in Australia and continue to be subjected to exclusion and persecution through the criminal justice system (Cunneen 2009: 210). In late 2019, Indigenous people represented 28% of the overall prison population despite only representing approximately 3–4% of the total population (Australian Bureau of Statistics 2019; Australian Institute of Health and Welfare 2019). As Rule, Brown and Ironfield (2021) suggest, such statistics show how incarceration operates as a ‘colonial function…to further the erasure of First Nations people’. Indigenous people also face institutional control through non-carceral mechanisms, for example, child protection and disciplinary measures targeting remote communities (Blagg and Anthony 2019: 162). Blagg and Anthony (2019: 162) explore how these non-carceral measures may be experienced ‘like a prison’ in a way that reduces the distinction between ‘inside’ and ‘outside’. As such, non-carceral and carceral mechanisms exist on a continuum that enables the discipline, punishment and control of Indigenous people, with incarceration as a primary tool.

Incarceration operates similarly for refugees. The *Migration Act (1958)*, as amended in 1992, establishes the mandatory detention of non-citizens without a valid visa. The period of detention need not be determined and can be indefinite. Immigration detention is not *officially* imprisonment, but immigration detention centres in Australia are inherently carceral spaces where refugees’ existence is controlled and their movement ‘bound’ (Pugliese 2002). The detention centres on Nauru and in Papua New Guinea are now officially closed, but as of 2019 refugees continued to be detained in accommodation in Papua New Guinea and Nauru with varying levels of ‘freedom of movement’ (Giannacopoulos and Loughnan 2020: 1127–1128; Grewcock 2017; Nethery 2019). Although refugees are no longer detained offshore in closed detention centres, these descriptions of restriction suggest that they are still subject to excessive controls that arguably amount to an ‘open air prison’ (Chomsky 2012).

As Giannacopoulos and Loughnan (2020) establish, carcerality as a form of control over refugees is firmly embedded within Australia’s settler-colonial logic where the carceral expansion of the Australian state is justified through the imagination of *terra nullius*. This colonial logic connects the forms of confinement enacted on refugees in Australia to the past and present histories of violence and dispossession against Indigenous people (Boochani 2020; Dehm 2020; Giannacopoulos and Loughnan 2020; Pugliese 2008). When these groups are detained in carceral spaces, the racist operation of necropower justifies extreme violence cloaked in the guise of legitimate punishment and discipline.

### Necropower as Justification for Violence

Necropower *justifiably* subjects refugees and Indigenous people in Australia to multiple forms of violence. This justification is deeply connected to the ‘enemy’ status imparted on these two groups and the associated rhetoric of ‘emergency’ and ‘exception’ (Mbembe 2019: 70). This rhetoric represents a continuation of the colonial project that silences marginalised voices as a form of epistemic and institutional violence (Bhatia 2020a; Nakata 2002; Spivak 1988). Examination of the rhetoric used to describe refugees supports this ‘enemy’ status. Refugees are described as ‘illegals’, ‘criminals’, ‘queue jumpers’, and potential ‘murderers [or] terrorists’ (Doherty 2015: 2), ‘alien…by virtue of citizenship’ (McHardy 2021: 13). Those who arrive by boat have additionally been legislated as an ‘illegal’ national security threat (Grewcock 2009, 2017) and framed as a threat to the very fabric of Australian society (Abbott 2015; Nethery 2019). This rhetoric ‘racially devalue[s]’ refugees (Bhatia 2020b: 278), makes it easier to expose them to racist violence that subjugates and harms (Bhatia 2020a, 2020b), and aligns with what Khosravi (2010: 126–127) terms ‘hostile hospitality’, whereby hospitality is reserved only for those migrants who are determined to be ‘good’ and ‘productive’ and, thus, ‘deserving’.

Indigenous people have similarly existed, and continue to exist, as a ‘fictionalized... enemy’ (Mbembe 2019: 70). The racist logic that accompanies this label justified the extreme violence and occupation of Indigenous people at the point of colonial invasion (Blagg and Anthony 2019: 170). Indigenous people also experience racism through the mechanism of ‘biocriminal’ where ‘criminality [is] conceptualized in racist terms’ (Foucault 1976: 258). As Cunneen (2009) establishes, Indigenous people are punished for crimes that are determined as such within Australian (white) law. Their subjugation is, thus, not considered an act of violence but merely a legal necessity (Anthony 2013: xi). Dodson (1994: 14) explores how this repression depends on the assumption that Indigenous people are ‘degenerates, drunks and criminals’ and ‘[t]o be otherwise…move[s] away from “Aboriginality”’. As Anthony (2013: 140) makes clear, this is a depiction that contemporarily influences criminal sentencing by leading to reduced empathy and increased ‘penality’.

It is well-established that racist logic leads to the actual death of Indigenous people and refugees, *including in carceral spaces* (Deathscapes 2020; Dehm 2020; Weber and Pickering 2011). What has not been explored in any detail is how necropower justifies multiple forms of violence, that operate within the carceral spaces of prison and immigration detention to produce a living death for Indigenous people and refugees.

### The Operationalisation of Violence Through Carceral Spaces

Immigration detention is an administrative control that is ‘*experienced as punitive’* and as a form of punishment (Bowling 2013: 300–301). As Pugliese (2008: 208) argues, because of the corresponding structures and experiences, the terms ‘detention centre’ and ‘prison’ *must* be recognised as synonymous. The violence of immigration detention is therefore clear and the uncertainty that arises from its indefinite nature is particularly cruel and psychologically distressing (Bhatia and Bruce-Jones 2021; Bosworth 2014: 183; Canning 2017: 71; Pugliese 2002). Reports of physical and sexual violence are also commonplace (Canning 2017: 71; Perera and Pugliese 2021; Vasefi 2021). Perera and Pugliese (2021: 69–72) rely on publicly accessible material to examine how ‘forced labour, sexual violence and border violence’ across detention facilities, works to ‘ravage the body, mind and soul’ of the refugees. This direct violence is also accompanied by ‘active neglect’, a term used by Loughnan (2019) to describe the intention of the state to cause pain when it backtracks on resettlement promises and fails to provide adequate medical care to refugees in detention.

There are also accounts of violence from the voices of those detained (Bhatia and Bruce-Jones 2021; Boochani 2018, 2020; Green et al. 2017). Boochani (2018) and Tofighian (2018, 2020) draw on Boochani’s experiences of detention on Manus Island and describe the violence there as a ‘kyriarchal system’. The term kyriarchal is employed here to depict immigration detention as a system that uses ‘systematic torture [to] erase the identity, agency and personhood of the imprisoned refugees’ (Tofighian 2020: 9). Boochani also reflects on the theme of time and explores how the uncertainty of indefinite detention is reproduced through various disciplinary processes in the daily lives of those imprisoned (Bhatia and Bruce-Jones 2021). As expressed by Boochani and Tofighian, unlike a camp, the system is not designed to kill but is instead ‘designed to [repeatedly] torture people’ (Bhatia and Bruce-Jones 2021: 82). This depiction is similarly expressed by Khosravi (2021: 13–14) who explores ‘waiting’ as an ‘experience of time’ that is hierarchically structured in accordance with intersecting and interlocking identities. In consistently failing to address, and also actively enabling, this violence, criminalisation and harm, Australia’s response to refugees must be characterised as state crime (Grewcock 2009; Vogl et al. 2020). Similar arguments have also been made about the experiences of Indigenous people in prison (Anthony 2018).

Indigenous people experience a specific ‘racialized violence…in the criminal justice system’ involving ‘physical violence, racist behaviour or verbal abuse’ that creates a ‘psycho-social domination…aimed at destroying the sense of self-worth through an attack on the person’s ethnic or racial background’ (Cunneen 2009: 220). Through their description of hierarchies that operate on racial terms within contemporary prisons, Blagg and Anthony (2019) show how this depiction of violence is fuelled by racism. They suggest that ‘exposure to death’ is clear in carceral institutions and refer to the torture and deaths examined by the Royal Commission into Aboriginal Deaths in Custody (1991), the multiple coronial inquiries since then, and the NT Royal Commission (2017) (Blagg and Anthony 2019: 171). Despite these ‘countless inquiries’, Blagg and Anthony (2019: 172) highlight that there has been no ‘criminal conviction’ of any of the ‘state officers responsible’. Referring to the NT Royal Commission (2017), Blagg and Anthony (2019: 189–190) explore the violence and harm experienced by Indigenous young people in prison as ‘a violation of the norm afforded to humanity’. Anthony (2018) additionally labels this harm a form of state crime that exists within continuing settler-colonial structures. These are structures that facilitate racial violence and the subjugation of Indigenous people (Anthony 2018; Johns 2018).

This violence that is experienced by Indigenous people in prison and refugees in detention is not merely coincidental, nor can it be attributed to wayward individuals (Anthony 2018). Violence and harm are built into the carceral architecture of immigration detention (Grewcock 2009; Pugliese 2008) and prisons (Carlton and Russell 2018; Johns 2018). Carceral spaces are by design intended to ‘sequester, discipline and punish’ and exist as ‘part of the discursive fabric that Foucault terms the ‘carceral continuum’’ (Pugliese 2008: 216). When Indigenous people and refugees are detained within these carceral spaces, the racist logic of the settler-colonial state justifies their subjection to multiple forms of violence (Mbembe 2019).

### Findings and Analysis

I qualitatively examine publicly accessible first-hand accounts from refugees in immigration detention and Indigenous young people in youth detention. Through this analysis, I demonstrate that refugees in immigration detention and young Indigenous people in youth detention experience four forms of violence: structural violence, epistemic violence, physical violence and brutality, and disciplinary violence. These four categories of violence are not easily separated and it is the overlapping and co-operation of these categories that creates a living death (Mbembe 2019).

### Structural Violence

Settler-colonialism and other intersecting systems of oppression are violent structures that cannot be untied from the incarceration of Indigenous people and refugees (Anthony 2018; Giannacopulos and Loughnan 2020; Rule et al. 2021; Tofighian 2020). Structural violence also plays out more narrowly within carceral spaces through harmful systems and processes. As shown in the first-hand accounts, structural violence existed within immigration detention and prison at a systems-level through, indefinite periods of detention, lockdown and isolation (Bhatia and Bruce-Jones 2021; Bosworth 2014; Canning 2017; Khosravi 2021), and inadequate medical care and inhospitable conditions that operated to cause pain and suffering (Loughnan 2019).

Turning first to indefinite detention, lockdown and isolation, the Indigenous young people and refugees explore how these structures and processes operated in cruel and harmful ways. For the refugees, indefinite detention caused pervasive feelings of uncertainty. Neda (Green et al. 2017: 152–153) explains that ‘you would fall in a deep, dark hole, because your life was in limbo and you were surrounded by the constant uncertainty in your life.’ A number of the refugees also explore how their perceptions of time were influenced by detention. Sajjd (Green et al. 2017: 241) describes that ‘it has reached a point that we don’t want to think about tomorrow, we don’t want to think about today’. Boochani (Green et al. 2017: 25) similarly explains that ‘the future – it’s too hard.’ These descriptions are usefully informed by Khosravi’s (2021: 14) depiction of ‘waiting’, where indefinite detention removes control and creates pervasive ‘uncertainty’ and ‘disconnectedness’.

Although the Indigenous young peoples’ sentences were all definite, harm from indefinite detention similarly arose because of the pervasive use of isolation and lockdown without clear release times. Isolation cells were used to punish, or when the young people were deemed to be a ‘risk’. Lockdown was used similarly. When the young people were on lockdown, they were only released from their cells for essential activities (BX 2017a, 2017b; CE 2017a). Lockdown and isolation are also forms of punishment that align with the pervasive use of confinement in Mettray, a carceral-style children’s home depicted by Foucault (1975: 293) as the ‘disciplinary form at its most extreme’. When the Indigenous young people were in lockdown and isolation, often they did not know when they would be released. During both isolation and lockdown, the Indigenous young people describe feeling stressed, sad and lonely (AF 2016, 2017; BE 2017a). AS (2017a: 6) further explains that ‘my brain went blank being in isolation…it was sort of like being a zombie’. Lockdown also had a disproportionately gendered impact. AX (2017a) recalls that there was only one young woman in detention when he was there and that she was locked down for a number of months so that she could be kept separate from the young Indigenous men. AX (2017a: 4) explains that ‘[s]he used to say how difficult it was being locked down by herself all the time.’

AN (2017a: 6), a young woman in detention, also recalls being kept in isolation for most of the time that she was in detention:

[e]very time I went in there I was never told how long I would be there. I would always ask and they would never tell me. They would say things like “fuck knows” and “how long is a piece of string”…The weirdest thing was that after all that time in there I ended up finding it really hard to be outside that cell.

This story suggests that the already problematic disciplinary mechanism of isolation is more oppressive if the isolation period is uncertain and extensive. AN (2017b: 13) further explains that ‘at the end of the day it’s them [the guards] walking home, not us, so we pretty much can’t do anything but sit there and get tortured’.

The Indigenous young people and the refugees similarly depict inadequate medical care and inhospitable living conditions (Loughnan 2019). The Indigenous young people and refugees were completely reliant on the prison guards and detention officers for medical care. As a result of deficient systems and processes, neither group had any control over when, or how, they obtained medical treatment. BF (2017a, 2017b) describes being initially refused medical treatment by a guard after notifying them that he had broken his collarbone. After he was finally taken to hospital and given a sling, he was told by some of the guards that the sling was a safety risk and was, therefore, required to remove it on multiple occasions. A few months after the initial break, he broke his collarbone again. BF (2017a: 8) explains that he was told to play sport, and notes ‘I felt like it wouldn’t have broken again if I hadn’t been told to play sport’.

Several of the refugees recall similar failings by the detention officers. Benjamin (Green et al. 2017: 50) remembers his father being airlifted to Darwin from Nauru after having a stroke and expresses dismay that ‘[a]fter two months they sent my father back here to Nauru. He was still the same. In that time, they didn’t do any medical checks for him’. Boochani (2018: 308) also recalls resorting to having the nerves in his tooth excruciatingly burnt off with a ‘red-hot wire’ by the Papua New Guinean guards, rather than attempting to receive appropriate care on Manus Island. This depiction of inaccessible medical treatment depicts a form of ‘active neglect’ (Loughnan 2019) that is similar to Boochani’s description of the medical care on Manus Island as a system that only keeps refugees alive just so that they can continue to be tortured by the structures of detention (Bhatia and Bruce-Jones 2021: 82).

The Indigenous young people and refugees also depict being forced to endure hot and unhygienic living conditions. AB (2017b: 47) notes that ‘[i]t was a hellhole…reeked of urine and shit. Mattresses were all dirty and mouldy.’ AB (2017a: 5) also describes constant ringworm infections from being forced to share ‘jocks’. AN (2017b: 11) similarly explains that ‘[t]here will be, like, dirty food on the ground, there will be spit, blood. Just brick. It’s dark with no light in there’. AS (2017a: 7) describes the ‘spit and snot on the walls as well as this red stuff which I am pretty sure was blood.’

As shown within the analysed accounts, the refugees also recall similar experiences when in immigration detention. Benjamin (Green et al. 2017: 53) refers to water shortages on Nauru and Peter (Green et al. 2017: 295) discusses food shortages in Scherger. Boochani (2018: 109, 121) similarly describes the ‘oppressively hot’ and crowded conditions in Manus Island, evidencing these conditions through his depiction of the bathroom (Boochani 2018: 160):

[t]he floor is always in the same state: piss up to the ankle…The toxic water has seeped into the surrounding area…It is such that anyone who wants to enter has to first go through a dense mass of weeds…that reach up to the waist.

He explains that the refugees would commonly prefer to relieve themselves in the bushes in the middle of the night (Boochani 2018: 161). These descriptions of the filthy cells and facilities, shortages of essentials, and exceptionally humid conditions, indicate the lack of control both groups had over their physical spaces. They were forcibly confined, with no way of improving these conditions without the approval of the authorities. When no approval came, the pain that the young people and refugees experienced simply had to be endured. In addition to enduring structural violence, the refugees and Indigenous young people also experienced a racist and degrading violence that operated to dehumanise.

### Epistemic Violence

Epistemic violence works to erase identities and devalue voices (Spivak 1988). In the first-hand accounts of immigration detention and prison, dehumanisation and humiliation existed as forms of epistemic violence. Many of the Indigenous young people recall being treated like animals, explaining ‘I felt like a little dog in a kennel’ (AK 2016: 4), ‘I was a caged animal, a monkey’ (AU 2017a: 6), and ‘[s]ome new guards treat us like dogs.’ (BC 2017a: 5). AN (2017a: 7) also states, ‘I wasn’t allowed a knife or a fork…I would have to scoop up my food with my hands…I felt like a dog.’ Dylan (2016a: 26) similarly describes that ‘I had no dignity and I wasn’t treated like a human being’.

The Indigenous young people were also repeatedly humiliated and insulted by the prison guards. They were called: ‘disgusting kid’, ‘scum’ (AN 2017a: 12), ‘[y]ou’re a waste of space’ (AS 2017a: 20), ‘bony cunt’ and ‘kid brain’ (BL 2017: 7). While other insults directly targeted race and included: ‘black cunt’ (AN 2017b: 19) and ‘dumb black kid’ (Jamal 2017a: 942). Many of the Indigenous young people recall feeling humiliated, defeated and sad after being insulted (BE 2017b; BQ 2017a). When the young people were psychologically distressed, these feelings were exacerbated. For example, AT (2017: 4) describes being marked ‘at risk’ when he tried to kill himself and explains that after this incident ‘[g]uards would walk past my room and tell me to kill myself. They break you down and keep telling you that you are shit, and then you start believing them.’ AN (2017b: 18) similarly describes wanting to self-harm after being told ‘that my mother doesn’t love me and…I should go and fucking kill myself, like I did plenty of other times.’ In this account, AN describes direct exposure to death through encouragement towards self-harm and suicide.

Nine of the young Indigenous people also recount, either themselves or others, being manipulated by the guards to fight each other. Often the young people were given chocolate, soft drinks or chips as a reward for engaging in the fights (AF 2016; AS 2017a, 2017b; AU 2017a, 2017b; AX 2017a; BR 2017a). AG (2017: 10) recollects that:

they turned us against each other and all that… the ones they didn’t like, they’d ask certain people, “Can you bash this person for me…”.

BE (2017a: 5) also recalls feeling ‘sad’ and ‘no good’ after one such fight, which resulted in the loss of a friendship with another young person in detention.

The young people were also humiliated by the guards in physical ways. AB (2017a: 15) explains that the young people were dared to ‘eat [horse] shit in return for chocolates and drinks from the guards’. Dylan (2016a: 27) recalls that a guard:

said to me, “*you can’t skull milk*”. I said I could. Then I was given a 1L carton of milk to skull. I didn’t know but he had tipped a lot of salt into the milk before he gave it to me. When I skulled the milk I almost vomited.

This behaviour, perpetrated by the prison guards, highlights an overarching culture of humiliation where physical and epistemic violence merged to induce feelings of shame. AQ (2017a: 11) explores the effects of such a culture when he describes being ‘made to feel useless and ashamed.’ Similar feelings of shame and despair were widespread among the accounts. Furthermore, the Indigenous young people commonly depict such dares, and the previously described insults, as racially targeted.

These depictions show how the dehumanisation and humiliation that the Indigenous young people experienced in prison resulted in shame, defeat and sadness and worked to ‘destroy’ their ‘sense of self-worth’ (Cunneen 2009: 220). These depictions tie in with Anthony’s (2018) description of the violence against Indigenous young people in youth detention as structural violence that is ‘not exceptional’ but that exists as part of a history of racist violence that has, and continues to, subordinate and dehumanise Indigenous people.

A number of the refugees recall similar feelings of dehumanisation and humiliation that often arose in response to the experience of indefinite detention. This sentiment of humiliation is shown by Amir (Green et al. 2017: 281) who explains that ‘for nothing I have been detained [and] humiliated for three years’. Boochani (Green et al. 2017: 9) also explores how the system ‘is trying to take people’s personalities by calling them by a number and humiliating them. After a long time they think they are not human.’ Boochani (2018: 232) highlights that ‘[t]here are so many times the prisoner is forced to straddle the border between human and animal.’ Hani (Green et al. 2017: 34) explores how:

most of the time, they changed your name into a number…By the time I finished eleven months, even if you call me Hani all the day, I would never say yes. If you called my boat number, I would say yes.

Peter (Green et al. 2017: 296) equally explains ‘I felt that I couldn’t use my name.’

Another common occurrence the refugees explore, is descriptions of people who ‘can’t remember their name’ (Green et al. 2017: 224). Sami (Green et al. 2017: 255) shares one such situation when he explains that ‘[t]oday my friend is mental, very mental…He lost his memory one year ago. He didn’t call his family, he doesn’t know who his family is.’ Similarly to the Indigenous young people, this complete disconnect between body and mind suggests that the inhuman treatment and violent structures faced by the refugees in detention operated, in some circumstances, to completely erase their sense of self. This is a brutality that also existed at a physical level.

### Physical Violence and Brutality

In the analysed accounts, the Indigenous young people and the refugees explore experiences of physical violence and brutality that rendered their bodies controlled and disposable (Mbembe 2019). Boochani (Green et al. 2017: 14) recalls the guards at Manus Island beating people, and Amir (Green et al. 2017: 279) remembers being pushed by a detention officer over an internet dispute. Abdul Aziz (Green et al. 2017: 230), Peter (Green et al. 2017: 294) and Boochani (2018: 90) refer to the use of beatings and tear gas as a response to acts of defiance or protest. Ariobarzan and Boochani similarly explore the physical violence they experienced when transferred from Christmas Island to Manus Island. Ariobarzan (Green et al. 2017: 249) explains, ‘[t]wo officers…held my hands firmly to prevent me from escaping. In that way, they took us to the plane. I didn’t know why.’ Boochani (2018: 96) asks, ‘[w]hat crime have I committed to justify cuffing me tightly and putting me onto an aeroplane?’. These stories show how physical violence, in the form of restraints, tear gas and beatings, was used by the officers to label the refugees as dangerous migrants ‘underserving’ of hospitality (Khosravi 2010).

Nineteen of the twenty-three Indigenous young people explore narratives that indicate the prison guards similarly used physical violence to assert their power in prison. This exertion of physical violence included forceful restraint (BC 2017b), the use of spit hoods (AB 2017b; BY 2017a, 2017b; Dylan 2016a), the use of restraint chairs, the use of painful restraint positions (Dylan 2016a) and the pervasive use of strip searches (AB 2017b: 50). Many of the young people also recall being tackled by multiple guards and ‘chucked on the floor’ (BV 2017a: 6, 2017b), grabbed by the throat and thrown down (BR 2017b), slapped (BW 2017; Dylan 2016a), choked (AX 2017b; BQ 2017b; Jamal 2017b) and given ‘wedgies’ (AX 2017b; BQ 2017a; Dylan 2016a). Dylan (2016b: 708) also refers to an incident where a number of the young people were tear gassed and recalls, ‘I thought I was going to die. My heart was racing because of the tear gas. My eyes were burning. I couldn’t hardly see properly.’

Any physical violence is shocking, but the vicious physical violence that was experienced by the Indigenous young people is differentiated by its brutality and ubiquitousness. The Indigenous young people describe cruel forms of punishment often delivered in front of others, where the body was not the ‘instrument or intermediary’ but the primary mechanism for punishment (Foucault 1975: 11). These mechanisms of violence are similar to the ‘crude displays of physical violence’ that racially targeted Indigenous bodies during colonisation (Anthony 2018; Blagg and Anthony 2019: 169). Thus, racism continues to result in brutal physical violence targeting Indigenous bodies. This brutality suggests that Foucault’s (1975) understanding of contemporary punishment, as directed at the soul and not the body, requires revision in the Australian settler-colonial context. In this context, racism justified the brutal targeting of both the body *and* mind (Mbembe 2019).

Two of the first-hand accounts also depict sexual violence. In the case of AN, a young Indigenous woman, her harm was perpetrated by the guards tasked with the responsibility of safeguarding her. In the case of Nima, despite being declared a refugee on the basis of his sexuality, and despite his increased susceptibility to harm while in offshore immigration detention, at no point was he moved onshore.

AN, one of the two young Indigenous women, recounts an experience of sexual violence. AN (2017a: 9) explains:

I clearly recall a time…when a large group of guards picked me up…It was mostly male guards and one or two female guards. They carried me into a room and threw me face down on a bed. They then used the Hoffman Knife to cut off all my clothes including my bra and underwear. I was fully naked and I felt real shame with all those men in the room. After a while of pinning me down they let me go and left the room. A short time later a guard opened the door and threw in an ‘at-risk’ gown.

This is a situation that is unimaginably horrific for any person but is made more disturbing as it was perpetrated by a group of ‘mostly male guards’ against a female child. While the account suggests the child was deemed to be a ‘risk’ to herself, it is impossible to imagine a situation where this assertion of force would justifiably ensure her safety. This account ties into Vasefi’s (2021) reports of sexual violence against women in immigration detention and Perera and Pugliese’s (2021: 72–73) description of Nauru Detention Centre, where the threat of sexual assault for women was commonplace and LGBTIQ + refugees were often exposed to the very conditions that they were seeking to escape.

Nima (Green et al. 2017: 182) identifies as a gay man. He informed the Australian authorities of this and explains that everyone knew of his sexual orientation on Nauru. Nima (Green et al. 2017: 182–183) explains that:

When I went to take a shower they [other detainees] were coming to open the bath door. They wanted to enter the bath by force and wanted to harass me. To sexually assault me… In detention there were tents…the tent was shared. When I fell asleep, other asylum seekers were harassing me, touching my buttocks and waking me from sleep. Always I was living in fear.

Despite this harassment, Nima was not provided with further protection but was simply warned that because homosexuality is illegal in Nauru he should not sleep in the same bed as his partner. Despite being declared refugees by the Nauruan government, the Australian state refused their requests to enter Australia, failing to give due regard to the health, safety and wellbeing of Nima and his partner. Sexual assault, thus, existed as a warped form of punishment that targeted their identities as gay men.

### Disciplinary Violence

The refugees and young people also explore disciplinary violence as forms of control and regulation that infiltrated into every aspect of their lives and operated to change behaviour through coercion and manipulation (Foucault 1975). The Indigenous young people describe constantly changing rules and a lack of clarity as to what the rules were. The young people were paralysed by the confusion this caused. Nine of the Indigenous young people do not recall the rules ever being formally explained. Others, like AB (2017a) and BV (2017a), describe a system in which rules changed constantly. BV (2017a: 3) further explains, ‘[i]t feels like you are always getting into trouble for breaking the rules because you do not know what the rules are or that they have changed.’ AX (2017a) and AT (2017) depict the lack of consistency between the guards as to rules. This lack of explanation created a constant atmosphere of stress and insecurity.

Many of the refugees similarly refer to rules and regulations that changed constantly and examine their excessive nature. Hal-Hal (Green et al. 2017: 115) suggests that they had ‘rules, like such crazy rules’, while Boochani (2018: 144) explains that they had ‘rules for both micro-control and macro-control’. Boochani (2018: 214–219) also suggests that the rules changed weekly and ‘hurl[ed] down on top of the prison, weighing down on the prisoner’s psyche.’ Imran (Green et al. 2017: 267) explores the consequences of these constant changes:

[o]ne day they say you can have an apple. The next day you can have two apples. The third day they say you can have three apples. Then on the fourth day, they say you can’t have an apple…And they stop giving us apples for a week. After a week, they start giving apples again. And everyone goes crazy about it…it’s the system that makes people crazy.

The constant rule changes caused anguish and operated to control and constrain. Hal-Hal (Green et al. 2017: 116) also explains that the rules often varied based on race:

[A] lot of people came after us but they transferred before, but all of them were white. They came from places like the Middle East, Lebanon, Iran. Plus, if you want to ask a Serco [guard] something…they will help people with white skin more than dark skin.

Hal-Hal’s account is similar to Blagg and Anthony’s (2019) depiction of racial hierarchies and shows that the refugees also experienced racism within immigration detention.

Boochani (2018: 124) also explores how disciplinary forms of control attempted ‘to turn prisoners against each other’. Boochani (2018: 196–197) describes how queuing, a simple mechanism that normally instils order, was used in detention to pit refugees against one another:

[t]he system is designed in such a way that people who enter the dining area ahead of others can eat more desirable food…But the people at the end of the line find nothing but junk left to eat… the queues have agency and they establish something: any person in the prison who behaves in a more despicable and brutish manner has a more comfortable lifestyle.

Aziz (Green et al. 2017: 227) explores similar disciplinary mechanisms when he explains that ‘[y]ou witness…some people fight over only small things. Why are people fighting because of just one spoonful of sugar?’. Hani (Green et al. 2017: 33) explores a more direct form of coercion when she discloses the following conversation she had with an immigration official:

[s]he was like, ‘Listen, you will never settle in Australia. Why did you come to this country? We don’t need people. If you want to come, go back and come by plane.’ And she was saying all these horrible things and everyone was crying.

In this example, the immigration official worked to change Hani’s self-perception, by influencing her to question her choices.

These descriptions of disciplinary violence are similar to Boochani and Tofighian’s depiction of the uncertainty of immigration detention operating alongside ‘other instructions or techniques of torture’ (Bhatia and Bruce-Jones 2021: 80). By operating alongside structural, physical and epistemic forms of violence, these disciplinary mechanisms impacted every aspect of the lives of the refugees and young people in a way that produced permanent suffering (Mbembe 2003).

### Living Death

In analysing these first-hand accounts, I explore how structural violence, epistemic violence, physical violence and brutality, and disciplinary violence, coalesce and interlock to create a web of ‘unfreedom’, ‘pain’ and suffering (Mbembe 2019: 91). Extreme forms of violence are used to dominate and control every aspect of the lives of the Indigenous young people and refugees. By targeting the refugees and Indigenous young people through structural, epistemic, physical and disciplinary mechanisms, the loss of ‘home’, ‘body’ and freedom over person and mind is assured (Mbembe 2019: 74–75). In response to these forms of violence, a recurring subject that the Indigenous young people and refugees discuss is self-harm, suicide and psychological distress, incidents that were produced by the harsh and violent conditions in prison and immigration detention (for example AN 2017a: 8; AT 2017: 3; Dylan 2016a: 21; Green et al. 2017: 52, 207). In such conditions, actual death or a violent marking of the body may disturbingly exist as one of the few ways freedom can be regained (Mbembe 2019: 91–92; Pugliese 2004: 28).

## Conclusion

In this article, I have examined how structural violence, epistemic violence, physical violence and brutality, and disciplinary violence, overlap and coalesce. All four mechanisms, exist as part of the ongoing structural violence of settler-colonialism, operate epistemically to erase identities, oftentimes operate in physical ways, and, work disciplinarily to control and regulate. By operating on multiple and overlapping levels, these mechanisms produce an experience of *living death* for young Indigenous people in youth detention in the Northern Territory and refugees detained in immigration detention. I suggest that this living death occurs because necropolitics *justifies* exposing refugees and Indigenous people to extreme forms of violence, harm that is *operationalised* through the violent, but seemingly legitimate, use of penitentiary techniques within the carceral spaces of prison and immigration detention.

What this article has not explored is whether these forms of violence are within the scope of Australian law. I do not present a comprehensive legal analysis, but what is clear is that the violence depicted in this article encompasses both legal (for example, Minister for Immigration and Multicultural and Indigenous Affairs v Al Khafaji’ (2002); ‘Al Kateb v Godwin’ (2004)) and illegal (for example, ‘Binsaris v Northern Territory’ (2020); ‘LO v Northern Territory’ (2017)) forms. As the acceptable level of punishment is lowered within carceral spaces (Foucault 1975: 302), the relevance of what is legal begins to wane. Regardless of law and regulation, intersecting and cruel forms of state-sponsored violence are experienced by Indigenous young people and refugees within carceral spaces and more easily depicted by the Australian state as ‘natural and legitimate’ (Foucault 1975: 302). Criminological frameworks that shift away from the state’s own legal systems are therefore crucial if criminologists are to effectively challenge this violence that is evidently state crime (Anthony 2018; Green 2006; Grewcock 2009). By setting out the voices of the Indigenous young people and refugees in the context of living death, the terror, pain and unending suffering of this violence is undeniable (Mbembe 2019). This article, thus, functions as a disruption to the societal tolerance to ‘punishment’ (Foucault 1975) and works to move beyond, as Gomeroi poet and researcher Whittaker (2020) terms, a ‘racist silence’ that we practice in Australia on a national scale.

Yet it is also important to acknowledge that I do not intend to label Indigenous people and refugees as ‘tragic and miserable victim[s]’ (Tofighian 2018: 365). Indigenous people and refugees continue to ‘refuse’ state violence (Blagg and Anthony 2019: 153) and claim self-determination over their own lives. McGranahan (2016: 320), as quoted in Blagg and Anthony (2019), suggests that this ‘refusal’ of state violence is:

often part of political action, of movements for decolonization and self-determination, for rights and recognition, for rejecting specific structures and systems.

All of the first-hand accounts in this article have been voiced in publicly accessible forums. By telling their stories publicly, they *refuse* inherently violent and state-created carceral systems. It is by exploring these stories through the lens of living death, that I re-iterate the need for criminologists to confront the state violence against Indigenous people and refugees that is *justified* by entrenched racism and *operationalised* by carcerality. In light of the prolonged detention of refugees in hotels and other immigration detention centres during the COVID-19 pandemic without appropriate health measures (Vogl et al. 2020) and the recent reversal of legal reforms that arose out of the NT Royal Commission (Fancourt and Havnen 2021), such challenges are desperately and urgently needed.

## Footnotes

1 I use the word Indigenous to refer to First Nations peoples, that is Aboriginal and Torres Strait Islander peoples in Australia.

2 When I refer to refugees I also refer to asylum seekers.
